# Dysregulated metabolic pathways in age-related macular degeneration

**DOI:** 10.1038/s41598-020-59244-4

**Published:** 2020-02-12

**Authors:** Meng Zhang, Nisi Jiang, Yi Chu, Olga Postnikova, Rency Varghese, Anelia Horvath, Amrita K. Cheema, Nady Golestaneh

**Affiliations:** 1 Department of Ophthalmology, Georgetown University Medical Center, Washington, DC 20057 USA; 2 Department of Neurology, Georgetown University Medical Center, Washington, DC 20057 USA; 3 Department of Biochemistry and Molecular & Cellular Biology, Georgetown University Medical Center, Washington, DC 20057 USA; 4 Laboratory of Retinal Cell & Molecular Biology (HNW28), NIH/NEI, Bethesda, MD 20814 USA; 5 Department of Pharmacology and Physiology, Department of Biochemistry and Molecular Medicine, George Washington University, Washington, DC 20037 USA; 6 Department of Oncology, Georgetown University Medical Center, Washington, DC 20057 USA

**Keywords:** Metabolomics, Mechanisms of disease

## Abstract

Age-related macular degeneration is a major cause of vision impairment in the Western world among people of 55 years and older. Recently we have shown that autophagy is dysfunctional in the retinal pigment epithelium (RPE) of the AMD donor eyes (AMD RPE). We also showed increased reactive oxygen (ROS) production, increased cytoplasmic glycogen accumulation, mitochondrial dysfunction and disintegration, and enlarged and annular LAMP-1-positive organelles in AMD RPE. However, the underlying mechanisms inducing these abnormalities remain to be elucidated. Here, by performing a comprehensive study, we show increased *PAPR2* expression, deceased NAD+, and SIRT1, increased PGC-1α acetylation (inactive form), lower AMPK activity, and overactive mTOR pathway in AMD RPE as compared to normal RPE. Metabolomics and lipidomics revealed dysregulated metabolites in AMD RPE as compared to normal RPE, including glycerophospholipid metabolism, involved in autophagy, lipid, and protein metabolisms, glutathione, guanosine, and L-glutamic acid, which are implicated in protection against oxidative stress and neurotoxicity, further supporting our observations. Our data show dysregulated metabolic pathways as important contributors to AMD pathophysiology, and facilitate the development of new treatment strategies for this debilitating disease of the visual system.

## Introduction

Age-related macular degeneration (AMD) remains the leading cause of blindness among the elderly in the developed world. More than 11 million people are affected in the US^1^ and AMD prevalence is anticipated to double by 2050^2^. Retinal pigment epithelium (RPE) cells are impaired in AMD and their dysfunction results in photoreceptor degeneration^3^. AMD is a multifactorial disease^2^ with complex underlying mechanisms. Studies suggest the contribution of genetic, environmental, and metabolic factors in AMD^4^. Drusen, the hallmark of dry AMD^5^ are extracellular deposits beneath the RPE that contain lipids and proteins.

Autophagy is known as a lysosome-mediated degradation process for damaged cellular organelles, providing energy to the cells^6,7^. Recently studies have associate autophagy to AMD^8,9^, and we have shown that RPE of AMD donors exhibit dysfunctional autophagy pathway^10^. We also reported decreased mitochondrial activity and disintegrated mitochondria, increased lipid droplets and glycogen granules, enlarged and annular autophagolysosomes, and increased cytotoxicity to oxidative stress^10^. However, the underlying mechanisms inducing these defective metabolic homeostases leading to AMD remain elusive.

The peroxisome proliferator-activated receptor-gamma coactivator (PGC)-1alpha (PGC-1α) is proposed as a master regulator of mitochondrial biogenesis^11^, respiration, adaptive thermogenesis, and multiple metabolic processes^12^. It is reported that PGC-1α affects cellular oxidative capacity and fatty acid oxidation through the regulation of mitochondrial and nuclear genes^13,14^. PGC-1α is also shown to regulate cellular mitochondrial content through biogenesis and degradation by autophagy/mitophagy^15^, and to play a role in lysosomal lipid trafficking^16,17^. PGC-1α repression is associated with various disorders, including diabetes, obesity, cardiomyopathy, and neurodegeneration^18–20^. In parallel, PGC-1α controls detoxification of oxidative stress by upregulating the expression of enzymes that scavenge ROS^21,22^. Ectopic cellular expression of *PGC-1α* is shown to improve survival during oxidative stress conditions. Conversely, the reduction of *PGC-1α* expression has been associated with sensitivity to oxidative stress^21^. PGC-1α activity is regulated by two main factors AMP-activated protein kinase (AMPK) and NAD+ -dependent deacetylase, SIRT1, that control cellular energy expenditure^18^. Interestingly, *PGC-1α* is highly expressed in retina^23^.

SIRT1 is a protein that regulates transcription silencing and is shown to expand life span in yeast, worm, and flies^24^. Studies have reported that Sirt1 activation in mice could promote healthy aging and protect from cancer^25,26^. SIRT1 can directly bind to transcription factors, including PGC-1α, and regulate their activity^18^. SIRT1 activates autophagy via deacetylating autophagy proteins^27^, and mitochondrial biogenesis through deacetylating and activating PGC-1α^28,29^. Reduced SIRT1 activity translates in increased PGC-1α acetylation and affects mitochondrial biogenesis and turnover.

The energy sensor, AMPK, is shown to regulate the level of SIRT1 co-substrate, the nicotinamide adenine dinucleotide (NAD+). AMPK and SIRT1 can regulate each other; they also target common molecules^30^. AMPK activation is induced through an increased AMP/ATP ratio in the cells^31^. Dysfunctional AMPK could result in reduced autophagy dynamics, which could trigger the accumulation of lipids and cellular wastes. It could also cause PGC-1α inactivation resulting in reduced mitochondrial biogenesis and turnover, which in turn could induce mitochondrial dysfunction.

PARPs constitute a large family of enzymes, catalyzing the transfer of ADP-ribose from NAD+ to receiver proteins^32^, and are shown to play important roles in crucial cellular functions^32^. PARP1 and PARP2 are the most characterized members of the PARP family of proteins^33^. PARP2 can directly bind to the SIRT1 promoter and negatively regulate its expression^34^. It has been reported that *Parp2* deletion in mice could increase Sirt1 levels, promote energy consumption, and augment mitochondrial biogenesis by deacetylation and activation of Pgc-1α and increase oxygen consumption^34^.

The mammalian target of rapamycin (mTOR) signaling pathway is a sensor of cellular stress and growth factor signals that regulates cell growth^35^ and inhibit autophagy^36,37^. mTOR consists of mTORC1 and mTORC2 complexes, which bind to different proteins and exert distinguished functions. mTORC1 contributes to protein translation, metabolism, and protein turnover, and mTORC2 is responsible for cell growth and migration^38^. Impaired mTOR activity has been associated with neurological disorders and neurodegenerative diseases^39^.

PGC-1α is shown to regulate normal and pathological angiogenesis in the retina^23,40^, inducing oxidative metabolism and antioxidant capacity in RPE^41^, and light sensitivity in the retina^23^. Here we sought to investigate the molecular mechanisms of AMD leading to dysfunctional autophagy and reduced metabolic activity in RPE. Our RNAseq data showed increased *PARP2* expression that acts upstream of SIRT1/PGC-1α and negatively regulates SIRT1. We also analyzed the AMPK/SIRT1/PGC-1α and mTOR activity and NAD+ levels, the expression of mitochondrial genes, and metabolic alterations in RPE cultured from AMD and normal donors and observed dysregulated metabolic pathways in AMD RPE.

## Results

### Increased *PARP2* expression, decreased NAD+, dysfunctional AMPK/SIRT1/PGC-1α pathway in AMD RPE

**Figure 1:**
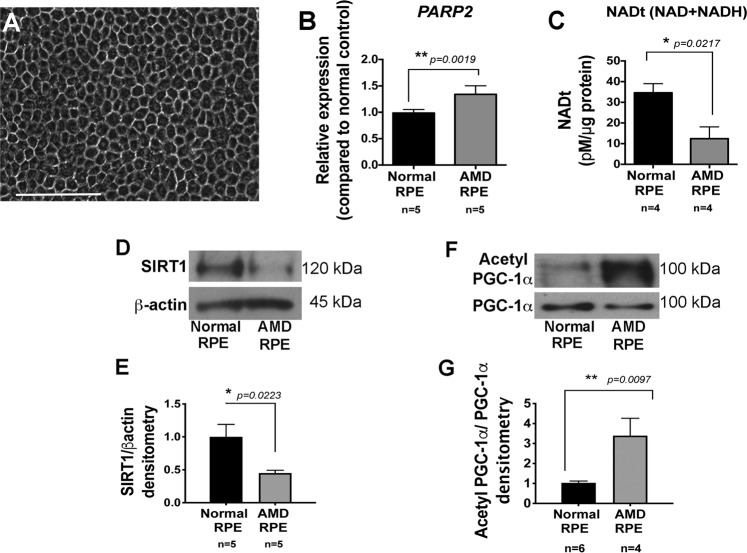
Increased *PARP2*, decreased NAD+ and dysregulated SIRT1/PGC-1α pathway in AMD RPE. (**A**) A representative image of human RPE monolayer culture from donor eyes (phase contras). (**B**) *PARP2* expression was significantly increased in AMD (n = 5) *vs*. normal RPE (n = 5). (**C**) Total NAD (NADt) levels were reduced in AMD RPE (n = 4) as compared to normal RPE (n = 4). (**D**) Representative western blot analysis of SIRT1 protein levels in AMD RPE (n = 5) as compared to normal RPE (n = 5). (**E**) Densitometry analysis of blots using ImageJ software showing significantly reduced SIRT1 levels in AMD RPE as compared to normal RPE. (**F**) A representative image of Immunoprecipitation (IP) with anti-acetyl lysine antibody followed by revealing with anti-PGC-1α antibody. Western blot analyses showed increased PGC-1α acetylation in AMD RPE (n = 4) as compared to normal RPE (n = 6). (**G**) Densitometry analysis of the blots with Image J software showing the ratio of acetylated/total PGC-1α protein levels in normal and AMD RPE. Asterisks indicate statistically significant differences in relative expressions between AMD and control samples, as determined by ANOVA analysis followed by Tukey’s test (p < 0.05).

Isolated native RPE from normal and AMD deceased donor eyes were cultured for 4 weeks to obtain monolayer of confluent RPE cells, as explained in our previous work, prior to performing any experiments^10^ (Fig. 1A) (Supplementary Table 1). We performed RNA sequencing (RNAseq) on native AMD RPE and normal native RPE cultured from AMD and normal eye donors. RNAseq revealed that *PARP2* expression levels were increased in AMD RPE as compared to normal RPE (Supplementary Table 2). To further confirm our RNAseq data, we performed qRT-PCR on AMD RPE and normal RPE. Our qRT-PCR data supported the RNAseq results and showed a significant increase in *PARP2* expression in AMD RPE compared to normal RPE (Fig. 1B). We also measured NAD+ levels in normal and AMD RPE. Our data showed a significant decrease in total NAD (NADt) levels in AMD RPE compared to normal RPE (Fig. 1C). We then evaluated the SIRT1 levels using western blot in normal and AMD RPE. Western blot, followed by densitometry analysis, revealed a significant decrease in SIRT1 protein levels in the AMD RPE as compared to normal RPE (Fig. 1D,E). Since SIRT1 deacetylates and activates PGC-1α, to test SIRT1 reduced activity, we measured the PGC-1α acetylated protein levels in native AMD RPE, as compared to normal RPE by immunoprecipitation (IP). IP followed by densitometry analysis revealed increased PGC-1α acetylated (inactive) form in native AMD RPE as compared to normal native RPE (Fig. 1F,G), further supporting our observations.

**Figure 2:**
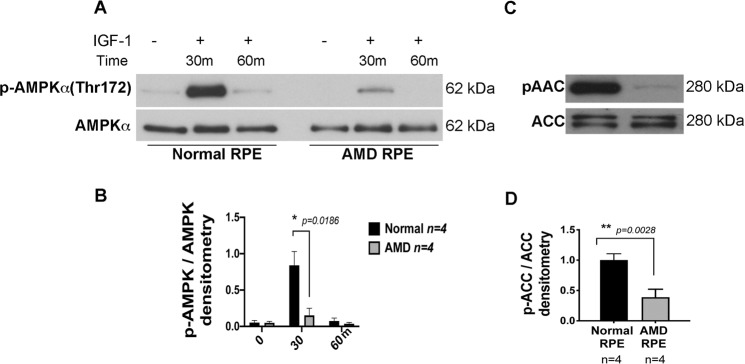
Dysfunctional AMPK pathway in AMD RPE. (**A**) A representative western blot image of phospho AMPKα(Thr172), in normal (n = 4) and AMD RPE (n = 4) cells. (**B**) Densitometry analysis of blots showing decreased AMPK phosphorylation in AMD RPE at 30 min incubation with IGF-1. (**C**) Representative western blot analysis of phospho Acetyl CoA Carboxylase, a direct AMPK target, in AMD RPE (n = 4) as compared to normal RPE (n = 4). (**D**) Densitometry analysis of blots showing a significant decrease in p-Acetyl CoA Carboxylase levels in AMD RPE. The ratio of phosphorylated/total protein levels was calculated for each one of the four control, and AMD samples and mean values are presented with standard deviations. Asterisks indicate statistically significant differences in relative expressions between AMD and control samples, as determined by ANOVA analysis followed by Tukey’s test (p < 0.05).

To test the AMPK activity, we analyzed time-dependent phosphorylation of AMPK by IGF-1 and observed significantly higher AMPK phosphorylation at 30 min time point in normal RPE as compared to AMD RPE by western blot followed by densitometry analysis (Fig. 2A,B). To further test the AMPK activity, we also measured the Acetyl-CoA carboxylase (ACC), a crucial enzyme in the biosynthesis and oxidation of fatty acids that is a direct AMPK target^42^. Western blot using anti pACC antibody followed by densitometry analysis revealed a significant decrease in pACC in AMD RPE as compared to normal RPE (Fig. 2C,D).

Dysregulated AMPK/SIRT1 and PGC-1α pathway, which could directly affect mitochondrial metabolism and biogenesis, led us to evaluate the RNAseq data for the mitochondrial genes such as *MRPL16* and *PMAIP1* in the AMD RPE as compared to normal RPE. *MRPL16* (Mitochondrial Ribosomal Protein L16) is a protein-coding gene and plays a role in mitochondrial translation^43^. The *PMAIP1 (*Phorbol-12-Myristate-13-Acetate-Induced Protein 1), also known as Noxa, stimulates apoptosis by activation of caspases^44^. The RNAseq showed increased expression of *MRPL16* and *PMAIP1* in the AMD RPE as compared to normal RPE (Supplementary Table 2). The RNAseq also showed differentially expressed genes that regulate mitochondrial function, such as *MDH1* (Malate Dehydrogenase 1). *MDH1* is an enzyme that catalyzes the reversible conversion of oxaloacetate and malate using NAD+/NADH^45,46^. Real-Time PCR further confirmed the RNAseq data for *MRPL16*, *PMAIP1*, and *MDH1 and* showed a significant increase in the expression of these three genes in the AMD RPE as compared to normal RPE (Supplementary Fig. 1A,D,G). We also focused on the *MAPK3* (Mitogen-Activated Protein Kinase 3), a member of the MAP kinase family that is shown to play important roles in multiple cellular functions, including cell cycle progression, proliferation, differentiation and apoptosis in response to extracellular stimuli^47^. *MAPK3* was not significantly changed in the AMD vs. normal RPE based on the RNAseq data (Supplementary Table 2). However, Real-Time PCR confirmed decreased expression of *MAPK3* in the AMD RPE as compared to normal RPE (Supplementary Fig. 1J). To test whether the gene expression changes were translated into protein levels, we measured the MRPL16, PMAIP1 and MDH1 and MAPK3 protein levels in AMD vs. normal RPE. Western blot analyses followed by densitometry did not show a significant difference in these protein levels in normal RPE as compared to AMD RPE (Supplementary Fig. 1B–L). This could be due to various levels of post-transcriptional and post-translational regulations and a high variability for correlation for various genes. Nonetheless, a small variation in gene expression could induce metabolic and epigenetic changes in a cell. Thus, our observations of differential expression of the above genes could have metabolic and biological effects in the RPE cells.

### Overactive mTOR pathway in AMD RPE

**Figure 3:**
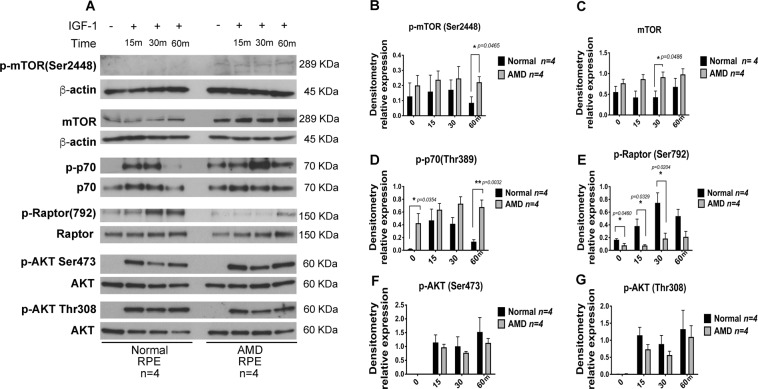
mTOR is overactive in AMD RPE. (**A**) A representative western blot of phospho mTOR, total mTOR, p70S6K, Raptor (Ser792), pAKT (Ser473), and pAKT (Thr308), in normal (n = 4) and AMD RPE (n = 4). **(B)** Relative expression of the phosphorylated mTOR(Ser2448), (**C**) total mTOR, (**D**) phospho p70S6K, (**E**) pRaptor(792), (**F**) pAKT (Ser473), and (**G**) pAKT (Thr308) were determined by densitometry analysis of the immunoblots. The ratio of phosphorylated/total protein levels was calculated for each one of the four control, and AMD samples and mean values are presented with standard deviations. Asterisks indicate statistically significant differences in relative expressions between AMD and control samples, as determined by ANOVA analysis followed by Tukey’s test (p < 0.05).

SIRT1 negatively regulates mTOR^48^, and we observed a decrease in SIRT1 levels and increased PGC-1α acetylation in AMD RPE. We, therefore, sought to test the mTOR pathway in AMD versus normal RPE. We measured the levels of mTOR phosphorylation at Ser2448, and the mTOR target protein^49^, the ribosomal protein S6 kinase (p70S6K) phosphorylation at Thr389, which indicates the mTORC1 activity. We also analyzed the levels of total mTOR protein in AMD as compared to normal RPE. Our data showed that phospho mTOR (Ser2448) was increased in AMD RPE as compared to normal RPE at 1 h after IGF-1 incubation (Fig. 3A,B). The mTOR total protein levels were also significantly higher in AMD RPE as compared to normal RPE (30 minutes IGF-1 incubation, Fig. 3A,C). The level of phospho p70 was induced by IGF-1 after 15 minutes incubation followed by a decrease after 30 minutes in normal RPE, in AMD RPE; however, the phsopho p70 was significantly higher in the absence of IGF-1 and was sustained until 1 h after incubation with IGF-1 (Fig. 3A,D).

To further investigate the mTOR hyperactivity and AMPK inactivity in AMD RPE, we analyzed Raptor (792) phosphorylation, which inhibits the Raptor-containing mTOR complex 1 (mTORC1), and is a direct substrate of AMPK^50^. Western blot analyses showed that the basal phosphorylation levels of Raptor (792) during starvation and in the absence of IGF-1 were higher in normal RPE as compared to AMD RPE. Furthermore, the addition of IGF-1 was able to induce a robust increase in phospho Raptor(792) levels up to 30 min, followed by a decrease after 1 h in normal RPE. In contrast, phospho-Raptor(Ser792) levels were significantly lower in AMD RPE in the presence of IGF-1, indicating a dysfunctional AMPK, and a constitutively active Raptor-mTOR complex (Fig. 3A,E). Since mTORC2 phosphorylates and activates AKT at Ser437, we tested the phosphorylation levels of AKT(Ser473) under the above conditions and did not see any significant difference in phospho AKT (Ser437) between AMD and normal RPE (Fig. 3A,F). Furthermore, AKT phosphorylation at Thr(308) was not significantly different between the AMD and normal RPE under the same conditions.

These observations indicate an overactive mTOR and dysfunctional AMPK pathway in AMD PRE cells and suggest that mTORC1 rather than mTORC2 is responsible for mTOR dysregulation in AMD RPE, thus, may be targeted to develop therapies for AMD.

### Metabolic dysregulation in AMD RPE

To investigate the metabolic dysregulation, we analyzed untargeted metabolomic and lipidomic profiling of AMD RPE and normal RPE cell extracts using high-resolution mass spectrometry. We acquired LC-MS data for 5 AMD samples and 4 normal samples. Each sample was run in triplicates, generating a total of 27 LC-MS spectra; however, technical replicates were not used for statistical analysis. The data were acquired in positive and negative ionization modes. The R-package XCMS was used to preprocess the datasets acquired in the electro-spray positive and negative ion modes and to detect the peaks.

2653 metabolite ions were detected in the positive mode, and 2542 ions were detected in the negative mode from the metabolomics profiling data.

Lipidomics profiling detected 1155 and 1699 peaks in the positive and negative ionization modes, respectively. Following data pre-processing, outlier screening was performed, and one AMD sample, and one normal sample were removed that behaved differently compared to other spectra as an outlier. A principal component analysis (PCA) analysis was performed on the preprocessed data. The score plots showing the similarities and differences between the metabolomics profiles of AMD and control are shown in Supplementary Fig. 3.

T-test was performed to identify ions with significant changes in intensity levels between AMD and control. Table 1 shows the number of significant ions selected for each analysis. As shown in Table 1, there were several ions that were significant with a p-value < 0.05, but very few were selected when adjusted for multiplicity using the Benjamini-Hochberg procedure for determining the false discovery rate at ≤5%.

**Table 1:** Number of ions selected by LC-MS.

Omics	ESI mode	^#^ions detected	p < 0.05	FDR < 0.05
Metabolomics	POS	2653	51	0
	NEG	2542	205	4
Lipidomics	POS	1155	99	2
	NEG	1699	266	0

**Figure 4:**
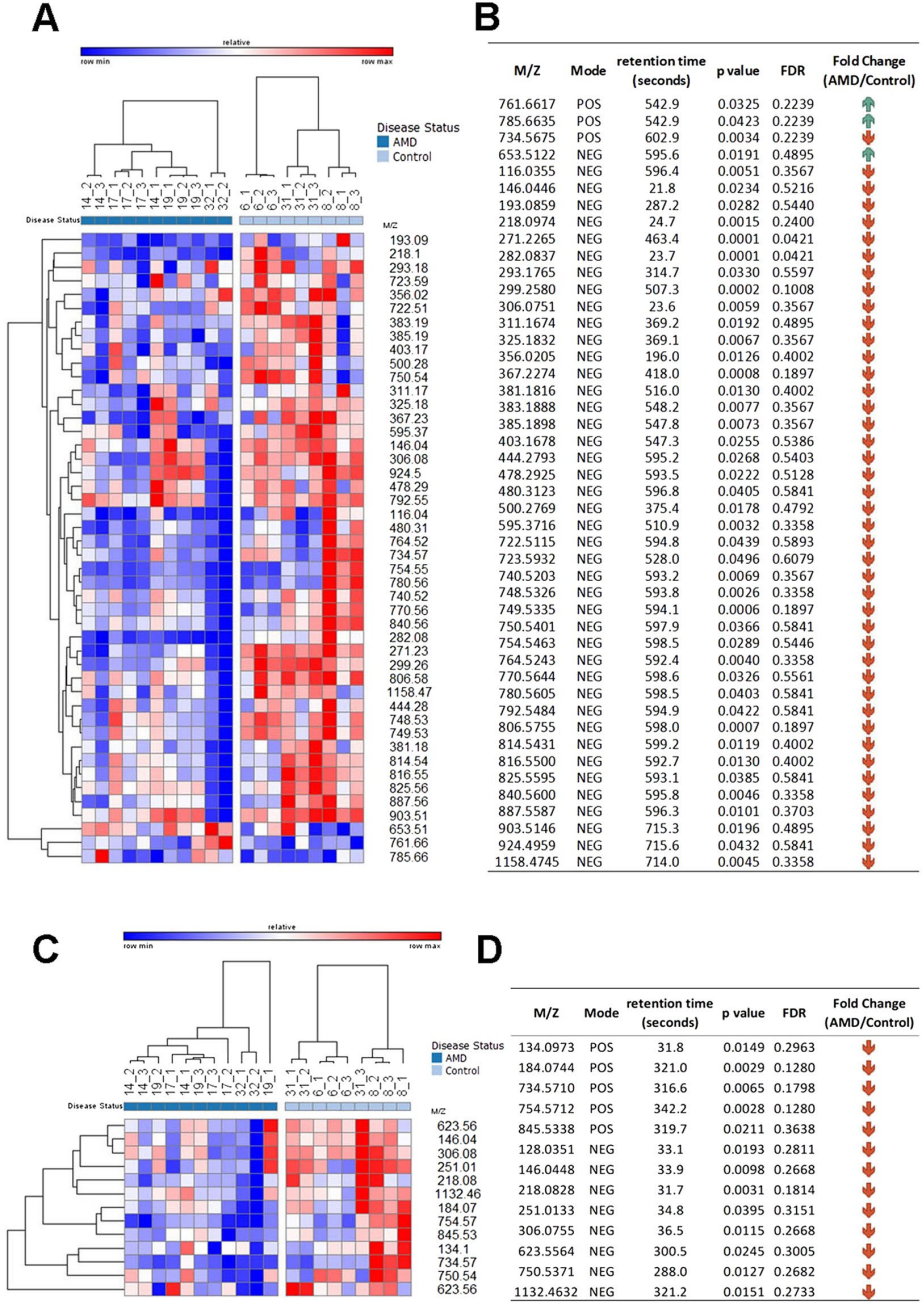
Heatmap of significant metabolites and lipids. (**A**) Relative expression of significantly dysregulated metabolites from LC-MS metabolomics analysis and (**C**) lipids from LC-MS lipidomics analysis. (**B**, **D**) are tables listing the masse over charge values, retention time, p-values, and direction of change for the selected metabolites in (**A**,**C**), respectively.

Putative identifications for the resulting ion list were obtained using the software MetaboQuest [http://omicscraft.com/MetaboQuest/], wich searched for putative identifications against multiple databases such as HMDB, METLIN, KEGG, MMCD, and LIPID MAPS. A mass tolerance of 10ppm was used to perform the database search. Pathway analysis was performed on a selected set of putative identifications that were filtered based on a literature search. Ions from positive and negative modes were combined for further analyses. 46 metabolites and 13 lipids with putative identifications were selected for further analyses after a literature search on the putative identifications. Figure 4A,B show the heatmap of these selected metabolites (4A) and lipids (4B). MetaboAnalyst^51^ was used to identify the pathways that are associated with the selected metabolites and lipids. From our analysis, a few metabolites such as glutathione, guanosine, and L-glutamic acid were found to be downregulated in AMD RPE as compared to normal RPE. The identity of these metabolites was confirmed by tandem mass spectrometry. MS/MS fragmentation patterns of the glutamic acid and glutathione against standards are shown in Supplementary Fig. 4. Dysregulation of these metabolites has been reported to be associated with oxidative stress and neurotoxicity^52–54^, cellular metabolism and stress resistance^55^, and recovery of mitochondrial membrane potential^56^; the findings by global MS profiling were strongly corroborated by our biochemical studies which showed a significant decrease in NADt in AMD RPE (Fig. 1C).

**Figure 5:**
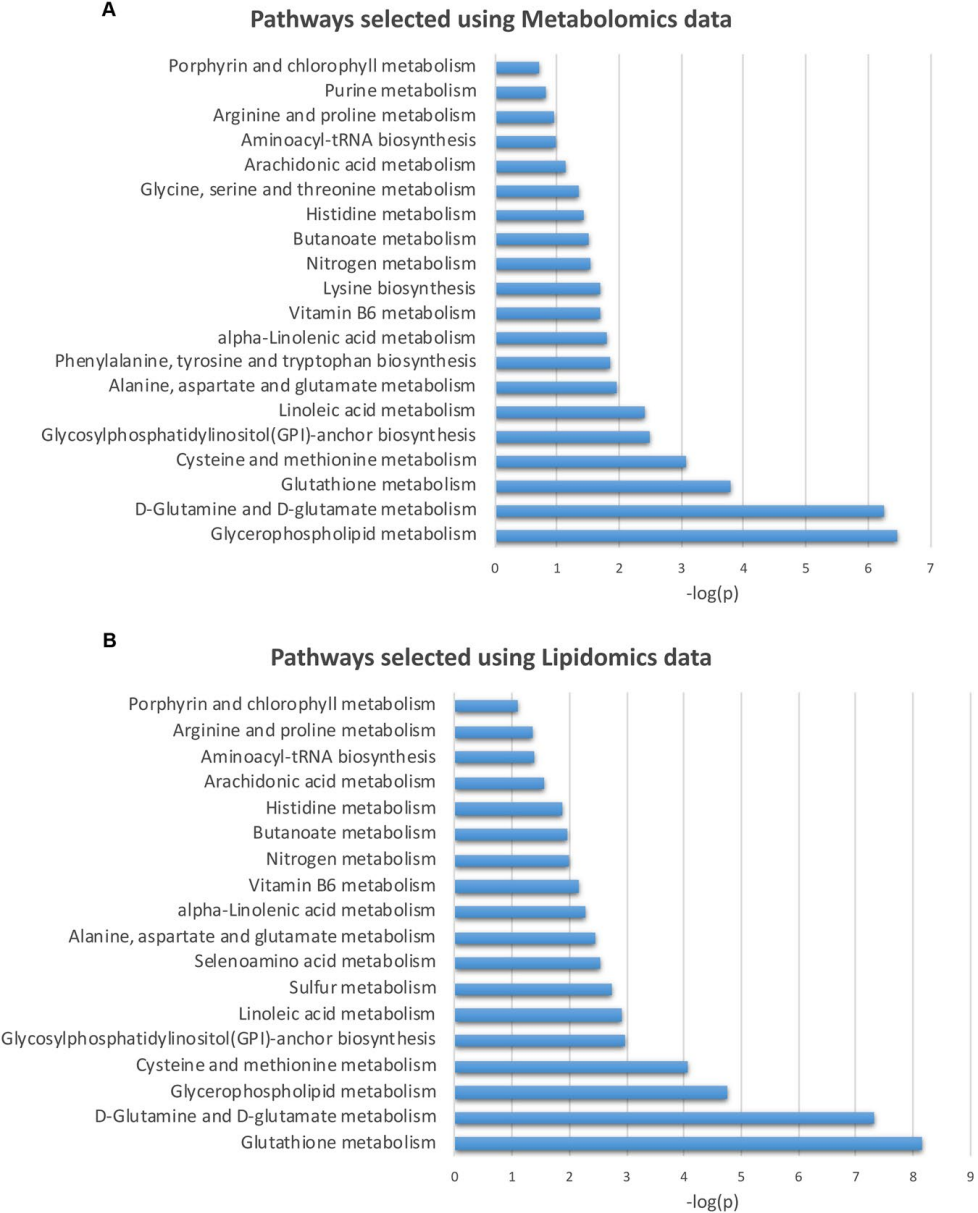
Pathway analysis using MetaboAnalyst. Pathways enriched in metabolomics (**A**) and lipidomics (**B**) data selected using MetaboAnalyst. The x-axis shows the −log(p) of the p-value calculated from the enrichment analysis. Since multiple pathways are evaluated in parallel, the statistical p values from enrichment analysis are further adjusted for multiple testing. There is a significant overlap between the pathways selected in both analyses.

Overrepresentation analysis with the hypergeometric test was used to determine if the dysregulated metabolites represent an enrichment of a particular pathway. Figure 5A,B represents the pathways selected for metabolomics (5A) and lipidomics (5B) data, which showed overlapping pathway enrichment for both sets of analyses. Interestingly, these metabolic pathways have been associated with AMPK/PGC-1α pathway in various studies^57–63^ and further support our observations on the implication of AMPK/SIRT1/PGC-1α and metabolic pathways in AMD.

Glycerophospholipid metabolism is significantly different in metabolomics and lipidomics data between AMD and normal RPE. The molecules involved in the glycerophospholipid metabolism pathway from the metabolomics and lipidomics analyses are Phosphatidylethanolamine (C00350), involved in the autophagy pathway, Phosphatidylcholine (C00157) involved in alpha-linolenic and linoleic acid metabolisms, 1-Acyl-sn-glycero-3-phosphocholine (C04230) and Phosphatidylserine (C02737) involved in lipid and protein metabolisms.

**Figure 6:**
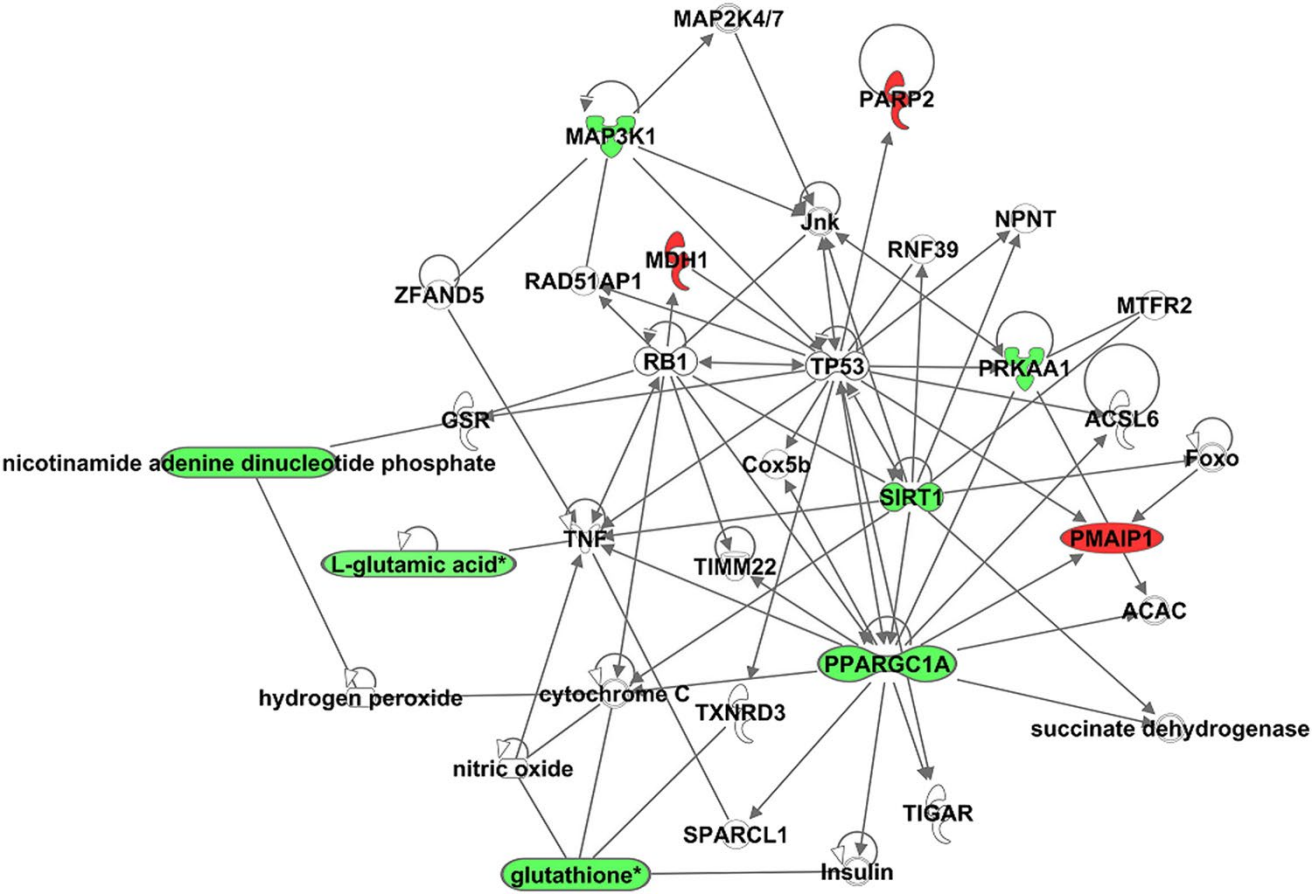
Combined network of genes, metabolites and lipids. The networks were generated by combining genes, metabolites and lipids through the use of IPA (QIAGEN Inc., https://www.qiagenbioinformatics.com/products/ingenuity-pathway-analysis).

To recapitulate our observations on gene expression, proteins, metabolomics, and lipidomics, we combined our data using the ingenuity pathway analysis tool and generated a combined network shown in Fig. 6, which showed a strong correlative network between metabolite indicators of oxidative stress with the expression of AMPK and Sirtuins.

**Figure 7:**
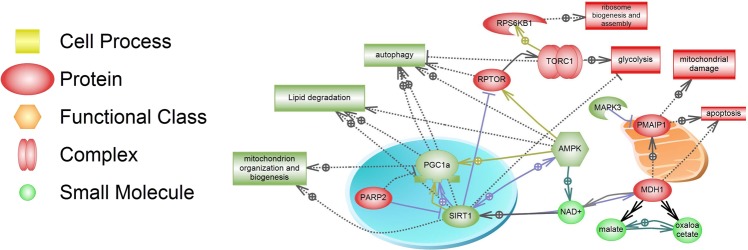
Crosstalk of metabolic pathways in AMD. Upregulation of *PARP2* expression in AMD leads to negative regulation of SIRT1. Depletion of SIRT1 protein, a NAD+ -dependent deacetylase, results in acetylation and inactivation of PGC-1α. SIRT1 downregulation leads to TORC1 complex activation in AMD that negatively regulates autophagy by phosphorylation-dependent inhibition of Atg13 and ULK1. Activated mTOR is also a positive regulator of glycolysis. *MAPK3* was downregulated in AMD RPE; which can result in higher expression of *PMAIP1* mRNA coding NOXA protein. This protein promotes mitochondrial membrane changes and efflux of apoptogenic proteins from the mitochondria. *MDH1*, an important gene that plays a role in energy metabolism was upregulated in AMD RPE cells. This cytosolic isozyme plays a key role in the malate-aspartate shuttle that allows malate to pass through the mitochondrial membrane. It also performs NAD/NADH-dependent reversible oxidation of oxaloacetate to malate. The activity of the energy sensor protein kinase, AMPK, was reduced in AMD RPE cells, which may lead to dysregulation of catabolic and anabolic processes, and to further downregulation of PGC1α and SIRT1. Proteins are shown according to their localization in the cell (nucleus, mitochondria, cytoplasm). Blue arrows indicate regulation of expression; green arrows indicate protein modification or binding; dash arrows show indirect regulation, and black arrows represent direct regulation. The green color of protein or cellular process indicates downregulation in AMD *vs*. normal RPE cells. The red color of protein or cellular process indicates upregulation in AMD *vs*. normal RPE. The pathway analysis was performed using Pathway Studio.

In addition, we used the Pathway Studio to generate a recapitulative pathway analysis of all metabolic pathways that are related to our observations and could be involved in AMD (Fig. 7).

## Discussion

In this study, we identified dysregulated metabolic pathways in AMD RPE as compared to normal RPE. We observed increased *PARP2* expression resulting in reduced NAD+ and SIRT1 and increased acetylated PGC-1α in AMD RPE. We also show reduced AMPK activity and hyperactive mTOR signaling in AMD RPE. These pathways are interrelated, and dysregulation of one will affect the other. Metabolomic analyses further revealed dysregulated metabolites and lipids contributing to oxidative stress and energy metabolism in AMD RPE as compared to normal RPE. Collectively, our study proposes dysregulation of AMPK/SIRT1/PGC-1α and overactive mTOR as underlying disease mechanisms in AMD RPE.

PARP2 is upstream of SIRT1 and inhibits SIRT1 translation, therefore regulating oxidative metabolism^34^. SIRT1 activity is NAD+ dependent, and a reduction in NAD+ can affect SIRT1 activity. We observed an increase in *PARP2* expression and decreased NADt in the AMD RPE compared to normal RPE. Increased *PARP2*, a consuming NAD+ enzyme, can inhibit SIRT1 levels, therefore lowering mitochondrial biogenesis through acetylation and deactivation of PGC-1α, which will result in decreased mitochondrial oxygen consumption^34^.

Concordant with our data, a new study showed that nicotinamide ameliorates disease phenotypes in an induced pluripotent stem cell model of AMD^64^. In addition, we observed decreased AMPK activity, as shown by decreased pAMPK and pACC levels in AMD RPE as compared to normal RPE. AMPK is a direct regulator of PGC-1α, and reduced AMPK activity could affect PGC-1α activity and expression. Recent studies report on the role of PGC-1α in regulating retinal angiogenesis in normal and pathological conditions^23,40^, as well as oxidative metabolism and antioxidant capacity in RPE^41^, and light sensitivity in the retina^23^. Nonetheless, the role of PGC-1α in AMD pathophysiology has yet to be discovered. Here we show that the PGC-1α acetylated form was increased in the AMD RPE as compared to normal RPE due to lower SIRT1 levels. Reduced PGC-1α activity could affect autophagy and mitochondrial biogenesis and consequently induce disease phenotypes that we observed in AMD RPE^10^.

Impaired mTOR activity has been involved in age-related diseases^39,65^, and neurological disorders, including neurodegenerative diseases^39^.

mTOR is shown to inhibit autophagy by phosphorylating ULK-1 and disrupting the interaction between ULK-1 and AMPK^37^. Another study reported that mTOR regulates autophagy termination and lysosomal reformation^66^. We have observed that phospho mTOR and total mTOR were increased in AMD as compared to normal RPE. We have also observed that mTOR target protein, p70S6K, is rapidly and sustainably activated by IGF-1 in AMD RPE compared to normal RPE, suggesting mTOR hyperactivity in AMD RPE. Furthermore, we found that Raptor (Ser792) phosphorylation, a direct target of AMPK, is inhibited in AMD, indicating repressed AMPK pathway in AMD RPE.

Relevant to the current study, altered mTOR signaling was reported in senescent RPE *in vitro*^67,68^. mTOR has also been shown to mediate the dedifferentiation of the RPE and initiate photoreceptor degeneration^69^.

mTOR is known to play a pivotal role in regulating lipid synthesis and storage, and inhibition of β-oxidation^70^. Furthermore, mTOR activation is associated with glycolysis and increased glycogen storage^71^. In our recent study, we analyzed AMD RPE by electron microscopy and observed increased lipid droplets and glycogen granules. We also observed that ATP was mainly generated by glycolysis in AMD RPE^10^. These observations are in line with our current observation of mTOR overactivity in AMD RPE.

It is reported that mTOR is required for the preservation of mitochondrial oxidative function through YY1-PGC-1alpha transcriptional complex^72^. While mTOR is crucial for mitochondrial biogenesis and activity^73^, an overactive mTOR inhibits AMPK and negatively impacts autophagy/mitophagy^36^. A recent study reported that AMD RPE are more resistant to oxidative stress after incubating the RPE under stress conditions for 24 hrs^74^. In our previous work, longer exposure to oxidative stress up to 48hrs showed that AMD RPE were more sensitive to oxidative stress-induced cell death as compared to normal RPE^10^. The same study showed that *PGC-1α* is increased in the RPE of AMD donors. This discrepancy could be explained by different AMD stages as RPE from donors with early AMD might first express higher *PGC-1α* levels as a compensatory response to excessive oxidative stress and due to higher mTOR activity^75^,  however, what is biologically relevant is PGC-1α activity. Our data show that acetylated PGC-1α (inactive form) is higher in AMD *vs*. normal RPE. Therefore, even in the presence of higher *PGC-1α* gene expression as a result of mTOR hyperactivity during early AMD stages, a reduction in PGC-1α activity in later stages could ultimately affect mitochondrial turnover and biogenesis causing increased ROS production and cell death.

AMPK is known to increase *PGC-1α* expression, and directly phosphorylates and activates PGC-1α^76^. AMPK also induces PGC-1α activation through SIRT1-mediated PGC-1α deacetylation^25^. Thus, a tight regulation between mTOR and AMPK pathways is necessary for PGC-1α regulation and function. An imbalance in the activity of these pathways can directly affect PGC-1α activity and consequently mitochondrial biogenesis and oxidative metabolisms, inducing AMD disease cellular phenotypes.

Retinal diseases, including AMD, are related to mitochondrial dysfunction^10,77,78^. Impaired mitochondria induce metabolic dysfunction, increased levels of ROS, and cell death^79^. Our data show that *MRPL16*, a protein-coding gene that plays a role in mitochondrial translation and *PMAIP1*, a protein that promotes activation of caspases and is implicated in mitochondrial damage and apoptosis, are upregulated in AMD RPE. In addition, our data revealed increased expression of *MDH1*, an enzyme catalyzing the transport of NADH throughout the mitochondrial membrane, and promoting reversible oxidation of oxaloacetate to malate^45^. These findings were consistent with the increase in metabolite indicators of oxidative stress. We also observed decreased expression of *MAPK3*, a gene that is involved in multiple cellular processes in a variety of extracellular signals. Interestingly, It is reported that *MDH1* can positively regulate *PMAIP1*^80^, whereas *MAPK3* inhibits *PMAIP1*^81^. These findings further support that AMD is a metabolic disease caused by mitochondrial damage and impairment in metabolic activities.

Downregulated AMPK/SIRT1/PGC-1α pathway and overactive mTOR led us to investigate metabolomics/lipidomics in the AMD RPE and normal RPE. Our data showed that the endogenous levels of specific lipids and metabolites are altered in AMD RPE as compared to normal RPE. Modifications in lipid metabolism could have multiple consequences including changes in gene expression and cellular functions, protein distribution and function and membrane compositions, which could lead to development of inflammation and various diseases^82^. Our metabolomic and lipidomic pathway analyses revealed certain pathways that were differentially expressed in normal vs AMD RPE. Interestingly, these pathways are associated with AMPK/SIRT1/PGC-1α pathway^57–63^, further supporting our observations. The glycerophospholipid metabolism pathway significantly differed in AMD and normal RPE (Fig. 5A,B). Interestingly, the phosphatidylethanolamine (PE) is one of two dominant glycerophospholipid classes in the vertebrate retina and the rod outer segment membranes and is involved in membrane transport of visual pigments^83^.

We combined significant metabolites and lipids with the genes that were selected from our RNA-Seq data and biochemical analyses to create a combined network using Ingenuity Pathway Analysis (IPA) tool (Fig. 6). The molecules in the network are mainly involved in cell functions, cell death and survival, neurological diseases, and organismal injury. The network analysis revealed that NADP selected from biochemical analysis and L-glutamic acid selected from the LC-MS analysis belong to the same Glutamate Biosynthesis II pathway. Glutathione^84^, guanosine^85^, L-glutamic acid^86^, and PPARGC1A (PGC-1α) are also involved in the depolarization of the mitochondrial membrane^56,87–89^ and the density of mitochondrial cristae^90^. The metabolites, reduced form of glutathione, and L-glutamic acid are found to be downregulated in our metabolomics and lipidomics analysis in AMD as compared to normal RPE.

Using Pathway Studio, we have recapitulated our biochemical and molecular analyses in Fig. 7.

In summary, our data provide strong evidence for the involvement of AMPK/SIRT1/PGC-1α and mTOR pathways in AMD RPE and propose dysregulation of metabolic pathways as underlying mechanisms in AMD. Our study provides new insights for the development of novel treatment strategies in AMD pathophysiology. While these *in vitro* analyses provide critical information for a better understanding of the underlying mechanisms of AMD, further *in vivo* analyses are required to confirm the *in vitro* observations.

## Methods

### Isolation and culture of RPE from deceased donor eyes

De-identified clinically diagnosed AMD (2 male and 3 female) and normal eyes (4 male and 3 female) were purchased from National Disease Research Interchange (NDRI, Philadelphia, PA, USA)^91^, a gift from Dr. Hathout, under an exempt IRB (NDRI). The eyes of donors with other ocular diseases or diabetes were excluded from the study. The average postmortem enucleation time was 9 h, and eyes were received in <24 h. NDRI performed serology tests and samples with infectious diseases were excluded (Table 1). RPE were isolated from the macula region according to established protocols^92^, and cultured in serum-free media^93^ with controlled oxygen (5%) and CO2 (5%)^94^. We then purified RPE by magnetic-activated cell sorting through positive selection with anti-BEST1 antibody (Abcam, Cambridge, MA, USA, 1/500) and anti-E-cadherin (Miltenyi Biotech, San Diego, CA, USA, 1/200); followed by a negative selection with anti-fibroblast MicroBeads (Miltenyi Biotech, 1/200) to eliminate fibroblasts^10,78^. To reduce heterogeneity, the highly epitheloid RPE colonies were manually selected and cultured.

All methods were carried out in concordance with relevant guidelines and regulations.

All experimental protocols were approved by a named institutional and/or licensing committee.

### Transcriptome sequencing and analysis

Whole RNA was extracted from RPE cells, and 5 ug was processed to generate sequencing libraries using the Ilumina TruSeq protocol. The library was subjected to paired-end sequencing (50 nt read length, 25 mln reads) on Illumina HiSeq2000 platform. Raw sequencer reads were processed using Illumina’s RTA and CASAVA pipeline software that includes image analysis, sequence quality scoring, and base calling. TopHat2 was used to align the quality-filtered sequencing reads. Sequences were aligned to the human genome reference genome, and transcripts were assembled using Cufflinks^95^. The abundance was evaluated as fragments per kilobase of exon per million fragments mapped (FPKM). Differential expression analysis is performed using the Cuffdiff utility of Cufflinks.

### Quantitative reverse transcription and polymerase chain reaction (qRT-PCR)

RNeasy Mini Kit (Invitrogen) was used to extract RNA. RNase-free DNase I was used for removing genomic DNA. Samples were then reversely transcribed using oligo-dT (SuperScript III cDNA synthesis kit from Qiagen). Quantitative Real-Time PCR was performed using the Power SYBR™ Green Master Mix (Applied Biosystems). Primers for each gene (Supplementary Table 3) were designed using PrimerQuest software (Integrated DNA Technologies).

### NAD measurement

Total NAD was measured with EnzyFluo NAD+/NADH Assay Kit (BioAssay Systems) based on the manufacturer instructions. Cells were lysed and homogenized in NAD extraction buffer. BCA assay (Thermofisher) was performed for the measurement of protein concentration and sample normalization. The standard curve for NAD was used by serial dilution, and 50 µl standard and cell lysate were used in a working solution. 96-well flat black plates (Greiner bio-one) were used, and fluorescence was read at λ_ex/em_ = 530/585 nm. Total NAD in samples was calculated based on the standard curve.

### Immunoblot analysis

Protein samples were extracted using radioimmunoprecipitation assay (RIPA) buffer containing 1% NP-40, 0.5% sodium deoxycholate, and 0.1% SDS, 150 mM NaCl, 50 mM Tris-HCl and freshly added Protease and Phosphatase Inhibitors (Invitrogen). Protein concentrations were determined using Pierce™ BCA Protein Assay Kit (Thermofisher). 30 μg of protein from each sample was used in NuPAGE gel electrophoresis and XCell Western blot (Invitrogen). Proteins were revealed using the primary and secondary antibodies according to the manufacturer protocol (Supplementary Table 4), and bands were revealed using the Clarity Chemiluminescent Substrate (Biorad) and captured with X-ray films. ImageJ software was used to perform densitometry analyses. For IGF-1 dependent phosphorylation analyses, the monolayer of AMD and normal RPE were cultured in serum free media and starved in HBSS for 2hrs followed by incubation with IGF-1 (75 ng/ml) for the indicated times.

### Image processing

Western blots are presented in raw format without any image processing. X-rays were scanned, and the Tiff files were color-inverted to gray scale. All raw images are presented in Supplementary Fig. 2, which includes all uncropped gels that were used to prepare Figs. 1–3, and Supplementary Fig. 1.

### Immunoprecipitation

Protein samples were extracted by Immunoprecipitation (IP) Buffer containing 25 mM Tris-HCl PH 7.4, 1% NP-40, 1 mM EDTA, 150 mM NaCl, and 5% Glycerol and freshly added Protease and Phosphatase Inhibitors (Invitrogen). Protein concentrations were determined using Pierce™ BCA Protein Assay Kit (Thermofisher). 150 μg Protein samples were incubated with anti-Acetyl Lysine Antibody conjugated-agarose beads (Immunechem) and incubated overnight at 4 °C. Agarose beads were then washed three times in IP buffer followed by three times wash in TAE buffer and incubated for 5 minutes at 95 °C in NuPAGE LDS Sample Buffer (Invitrogen). Supernatants were collected after brief centrifuge and were cooled at room temperature before loading. Western blots were revealed with anti-PGC-1α antibody (Millipore) to determine PGC-1α acetylated protein levels.

### Metabolomics and lipidomics

Metabolite extraction was performed using modification of the protocol described by Sheikh *et al*.^96^. Briefly, cells were harvested by removing the media and washing with PBS, followed by cell scraping. Metabolite extraction was performed using sequential extraction as described by Sheikh *et al*. The samples were reconstituted in 200 uL of 50% Methanol in water for UPLC- QTOF MS analysis (Xevo G2 QTOF-MS, Waters Corporation, USA). The data were acquired in electrospray positive and negative mode for maximizing metabolome coverage. LockSpray interface of Leucine- Enkephalin (556.2771 [M + H]+ or 554.2615 [M − H]−) at a concentration of 2 ng/µl in 50% aqueous acetonitrile was used for correction of the mass error in real-time. Additionally, protein quantification was performed using the Bradford method for sample normalization.

### Statistical analysis

Experiments were repeated at least 3 times. For gene expression analyses, 3 replicas of each sample were represented per experiment, and mean averages ± SEM were calculated. We performed one-way ANOVA followed by Dunnett’s multiple comparisons test using GraphPad Prism version 7.00 for Mac, GraphPad Software, La Jolla, California, USA. The statistical significance was determined as a p-value < 0.05.

For Metabolomics and lipidomics data analyses, centroided and integrated mass spectrometry data from the UPLC-TOFMS were processed using XCMS (Scripps Institute) to generate a data matrix containing ion intensities, mass to charge (m/z) and retention time values. Pathway analyses were performed using MetaboAnalyst 4.0 (PMID: 29762782), and the networks were generated using Ingenuity Pathway Analysis tools (QIAGEN Inc., https://www.qiagenbioinformatics.com/products/ingenuity-pathway-analysis).

## Supplementary information

Supplementary Data.

## Data Availability

The datasets generated during and/or analyzed during the current study are available from the corresponding author on reasonable request.
