# Supplementary material for: Dysregulated metabolic pathways in age-related macular degeneration
Source: Sci Rep. 2020 Feb 12;10:2464. doi: 10.1038/s41598-020-59244-4 (PMC7016007; doi:10.1038/s41598-020-59244-4)
Supplement: Supplementary file 1 — Supplementary Data. [file 41598_2020_59244_MOESM1_ESM.pdf]

Supplementary Data.

Dysregulated metabolic pathways in age-related macular degeneration.

Meng, Zhang[1], Nisi Jiang[1], Yi Chu[1], Olga Postnikova[4], Rency Varghese, Anelia Horvath[5], Amrita K Cheema[6,3], Nady Golestaneh*[1, 2, 3]

[1] Department of Ophthalmology, Georgetown University Medical Center, Washing DC 20057

[2] Department of Neurology, Georgetown University Medical Center, Washington DC 20057

[3] Department of Biochemistry and Molecular & Cellular Biology, Georgetown University Medical Center, Washington DC 20057

[4] Laboratory of Retinal Cell & Molecular Biology (HNW28), NIH/NEI, Bethesda, MD 20814

[5]Department of Pharmacology and Physiology, Department of Biochemistry and Molecular Medicine, George Washington University, Washington DC 20037

[6] Department of Oncology, Georgetown University Medical Center, Washington DC 20057

* Correspondence should be addressed to:

Nady Golestaneh, PhD, MSc.

Departments of Ophthalmology, Neurology, Biochemistry and Molecular & Cellular Biology,

Georgetown University Medical Center, 3900 Reservoir Road NW, Medical-Dental Building, Room NE203

Washington, DC 20057,

Phone: 202-687-4309,

Email: ncg8@georgetown.edu

Supplementary Table 1. RPE cultured from AMD and normal donor eyes

DONOR ID#	DONOR AGE-GENDER (F: FEMALE, M: MALE)	Clinical Diagnosis	CFH (C: RISK)	HTRA1 (A:RISK)	Smoking	Cause of death	Enucleation (hrs)
003	57-F	NORMAL	TC	AA	1ppd	Lung Cancer	10
006	72-M	NROMAL	CT	AG	Quit in 1993	Chronic obstructive pulmonary disease	12
008	48-F	NORMAL	TT	GG	No	Congestive Heart Failure	14
010	80-M	NORMAL	CC	AG	Quit in 1984	Acute Myocardial infraction	9.4
020	68-F	NORMAL	TT	GG	No	Chronic obstructive pulmonary disease	8
025	50-M	NORMAL	TT	GG	No	Myocardial infraction	17
031	49-M	NORMAL	TC	GG	A few packs per week	Myocardial infraction	9.5
009	68-F	AMD	TT	GG	2 ppd for 40 years	Stroke	3
014	82-M	AMD	CT	AG	1 ppd for 40 years	Cardial related	12
017	81-M	AMD	CC	AA	N/D	Cardial arrest	10.5
019	80-F	AMD	CT	GG	No	GI bleed	9
032	75-F	AMD	CT	AA	No	Pancreatic cancer	7

Supplementary Table 2. Next generation RNAseq analysis of specific genes in AMD *vs.* normal RPE.

Gene	Specification	Fold-changed AMD (n=5) *vs.* Normal (n=5)	*p*-value
PARP2	Poly(ADP-Ribose) Polymerase 2. Participates in DNA double strand repair and nucleotide excision repair.	1.35	0.027
MRPL16	Mitochondrial Ribosomal Protein L16. Involved in Mitochondrial Translation.	1.82	0.029
PMAIP1	Phorbol-12-Myristate-13-Acetate-Induced Protein 1. Related pathways are Apoptosis and Autophagy.	2.92	0.024
MDH1	Malate Dehydrogenase 1. Related pathways are Citrate cycle and Glucose metabolism.	1.25	0.029
MAPK3	Mitogen-Activated Protein Kinase 3. Involved in many biological functions including cell growth, adhesion, survival and differentiation.	1.026	0.94

Supplementary Table 3. Primer sequences

Gene	Primer Sequence	
GAPDH	Forward	GTCTCCTCTGACTTCAACAG
	Reverse	GTCTCTCTCTTCCTCTTGTG
PARP2	Forward	GTGGAGAAGGATGGTGAGAAAG
	Reverse	CTCAAGATTCCCACCCAGTTA
PMAIP1	Forward	GGAGATGCCTGGGAAGAAGG
	Reverse	ACTCGACTTCCAGCTCTGC
MRPL16	Forward	TGCAATCTTGGCATTGGGTG
	Reverse	GCTTGAAAGGGGCTGGTACT
MDH1	Forward	ACCAGGGCAGCGTAAACTAC
	Reverse	GGACTCTGATTGGTTCAGACATGA
MAPK3	Forward	TCAGCCGCTCCTTAGGTAGG
	Reverse	TCAGACTCCAAAGCCCTTGAC

Supplementary Table 4.

Name of Antibody	Concentration	Ref#
P70 S6 Kinase (49D7) Rabbit mAB	1:1,000	Cell Signaling, 2708<br>Fujiwara, Hiroaki, et al. *Scientific Reports* 9.1 (2019): 1-14.
Phospho-p70 S6 Kinase (Thr389) (108D2) Rabbit mAb	1:1,000	Cell Signaling, 9234<br>Nakajima, Shotaro, et al. *Scientific Reports* 9.1 (2019): 1-11.
AMPK-alpha (D63G4) Rabbit mAb	1:1,000	Cell Signaling, 5832<br>Xu, Weilin, et al. *Journal of Neuroinflammation* 16.1 (2019): 1-14.
Phospho-AMPK alpha (T172) (40H9) Rabbit mAb	1:1,000	Cell Signaling, 2535<br>Zuo, Zhi, et al. *Oxidative Medicine and Cellular Longevity* 2019 (2019).
Acetyl-CoA-Carboxylase 2 (D5B9) Rabbit mAb	1:1,000	Cell Signaling, 8578<br>Ma, Xueqiang, et al. *Molecular medicine reports* (2019).
Phospho-Acetyl-CoA Carboxylase (Ser79)	1:1,000	Cell Signaling, 3661<br>Su, Zhiduan, et al. *Nature communications* 10.1 (2019): 1-18.
Raptor (24C12) Rabbit mAb	1:1,000	Cell Signaling, 2280<br>Su, Zhiduan, et al. *Nature communications* 10.1 (2019): 1-18.
Phospho-Raptor (S792) Rabbit mAb	1:1,000	Cell Signaling, 2083<br>Danielpour, David, et al. *Scientific reports* 9.1 (2019): 1-17.
SIRT1 (D1D7) Rabbit mAb	1:1,000	Cell Signaling, 9475<br>Akhtar, Safia, and Helmy M. Siragy. *PloS one* 14.12 (2019).
Anti-PGC-1 Antibody mouse mAB	1:1,000	Millipore Sigma, ST1202<br>Koh, Y.J., et al. 2009. *Exp. Mol. Med.* 41, 880.
β-Actin (D6A8) Rabbit mAb	1:10,000	Cell Signaling, 8457<br>Luengo, Alba, et al. *Nature Communications* 10.1 (2019): 1-11.
Anti-Acetyl Lysine Antibody- Conjugated Agarose Beads	0.8µg/µl	Immunechem, ICP0388<br>Chunaram, Choudhary, Kumar Chanchal, and Gnad Florian. *Science* 325 (2009): 834-40.
Anti- MRPL16 Rabbit anti-Human Polyclonal (C-Terminus) Antibody	1:1000	Lifespan, LS-C354178-100
Anti-PMAIP1 (Noxa) Rabbit mAb Antibody	1:1000	Cell Signaling,14766<br>Ishikawa, Kazuma, et al. *Oncology reports* 42.6 (2019): 2416-2425.
Anti-MDH1 polyclonal Antibody	1:500	Fisher Scientific, PIPA586808
Purified Mouse Anti-ERK1 (MAPK3) Antibody	1:1000	BD Biosciences, 610408<br>Boulton TG, Cobb MH. Cell Regul. 1991; 2(5):357-371
Anti-Rabbit IgG HRP-Linked Antibody	1:3000	Cell Signaling, 7074<br>Yan, Jingyi, et al. *Frontiers in Cell and Developmental Biology* 7 (2019): 288.
Anti-Mouse IgG HRP-Linked Antibody	1:3000	Cell Signaling, 7076<br>Yan, Jingyi, et al. *Frontiers in Cell and Developmental Biology* 7 (2019): 288.

Supplementary Figure 1.

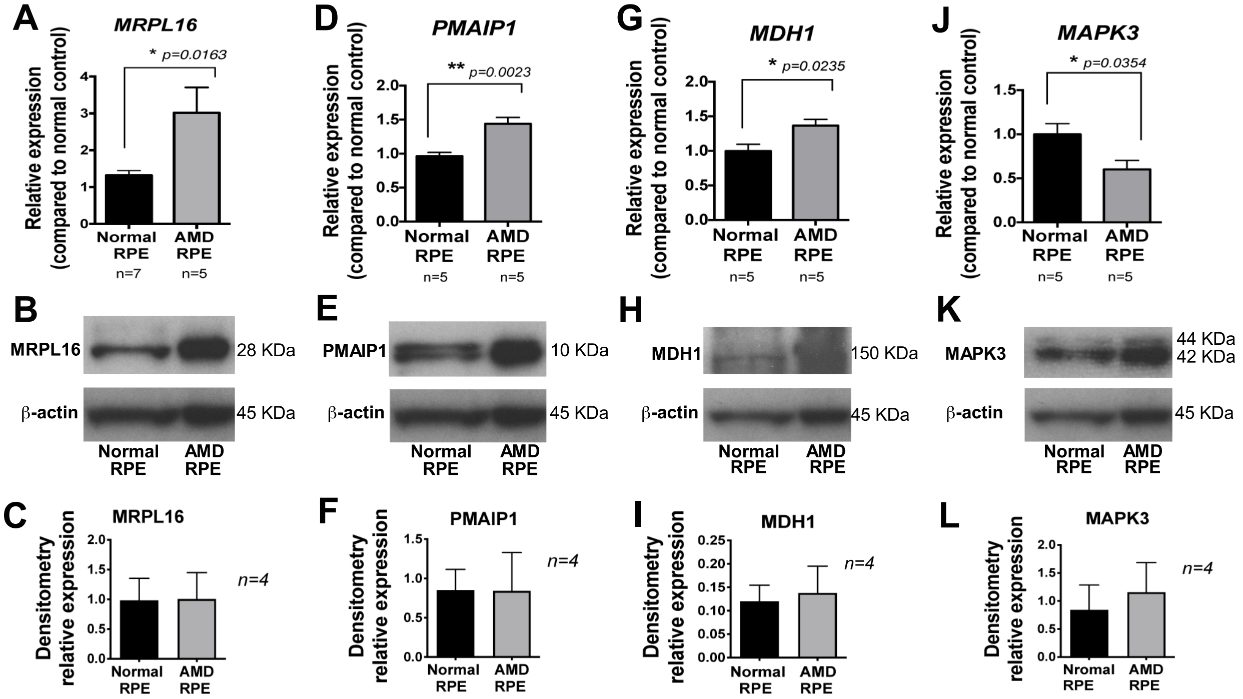

Mitochondrial genes and genes regulating mitochondrial functions are differentially expressed in the AMD RPE.

(**A**) *MRLP16* gene expression is increased in AMD RPE as compared to normal RPE measured by real time PCR, (**B**) a representative western blot for MRLP16 protein, and (**C**) densitometry analysis of immunoblots for MRPL16 protein expression. (**D**) *PMAIP1 gene* expression is increased in AMD RPE as compared to normal RPE, (**E**) a representative western blot for PMAIP1, and (**F**) densitometry analysis of immunoblots for PMAIP1 protein expression. (**G**) *MDH1* gene expression is increased in AMD RPE, (**H**) a representative western blot forMDH1 protein, and (**I**) densitometry analysis of immunoblots for MDH1. (**J**) *MAPK3* gene expression is decreased in AMD RPE as compared to normal RPE measured by real time PCR, (**K**)a representative western blot for MAPK3 protein expression, and (**L**) densitometry analysis of immunoblots for MAPK3.

Supplementary Fig. 2

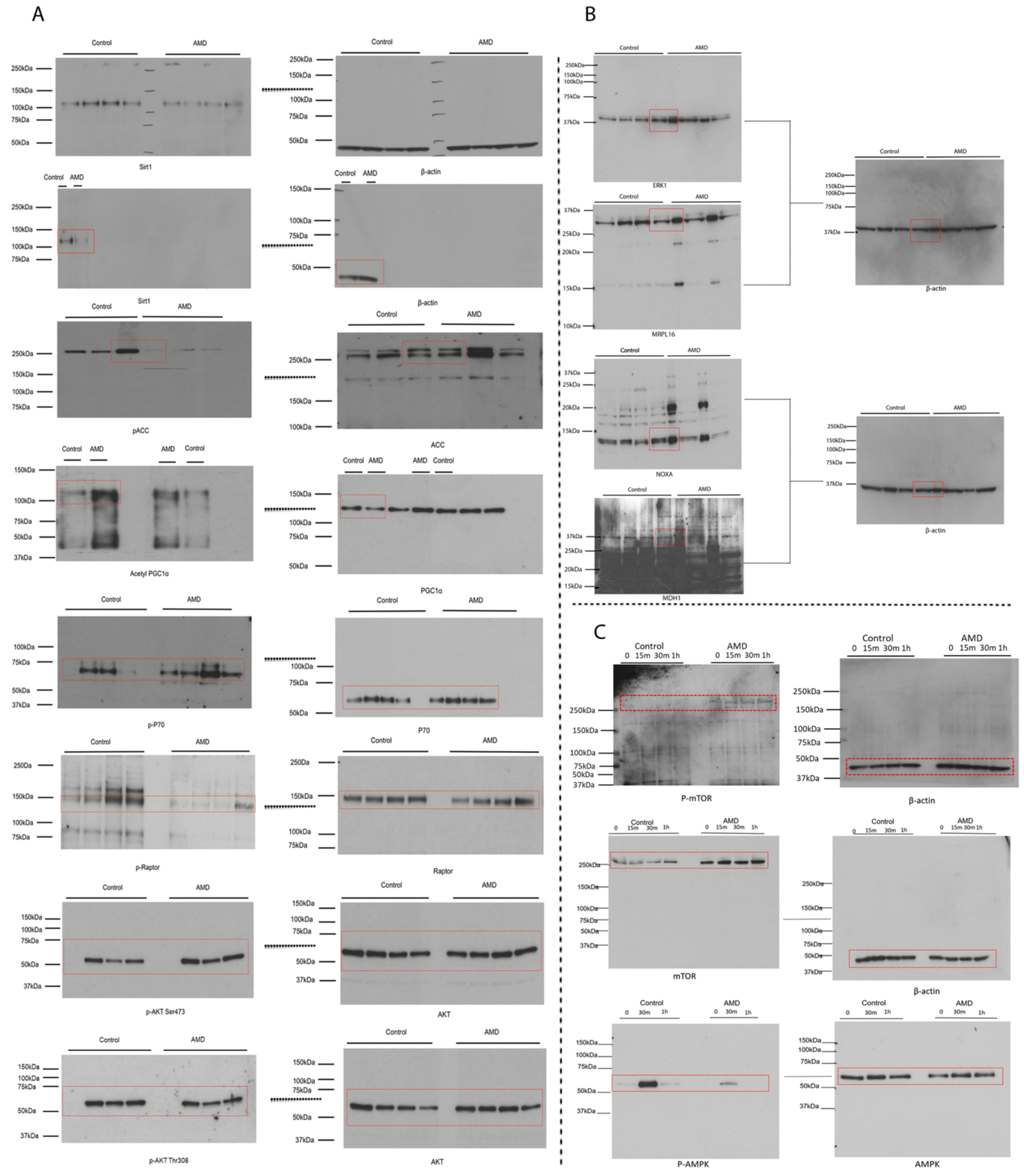

The uncropped images for western blots. Ladders on the left of each gel represent the molecular weight in kDa. Solid lines on the top of each gel partitions the grouping of the sample. Red boxes display the cropped area used in the main figures. Connectors between the two gels demonstrate the β-actin or internal control for the target protein.

Supplementary Figure 3

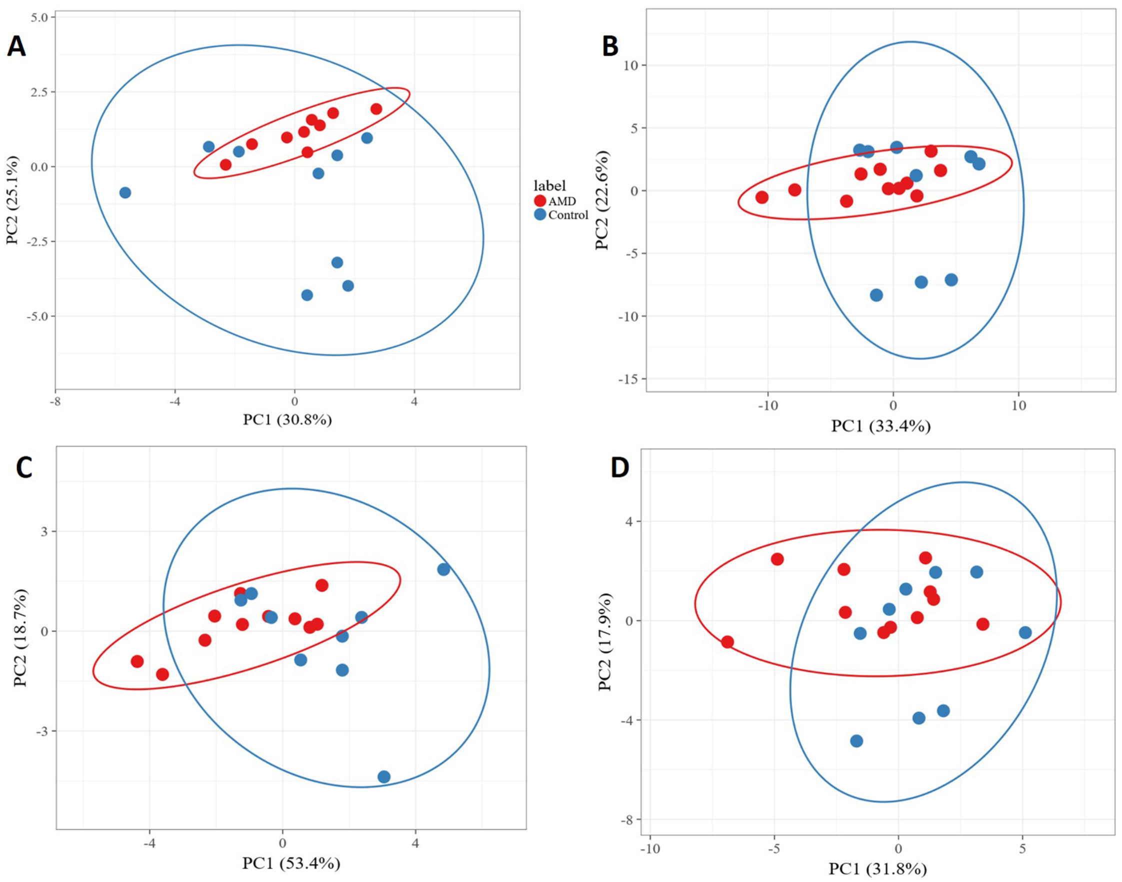

PCA scores plot of metabolomics and lipidomics data:

Principal component scores plot showing the similarities and differences between the metabolic profiles of AMD and Control in (A) metabolomics positive mode, (B) metabolomics negative mode, (C) lipidomics positive mode, and (D) lipidomics negative mode data.

Supplementary Fig. 4

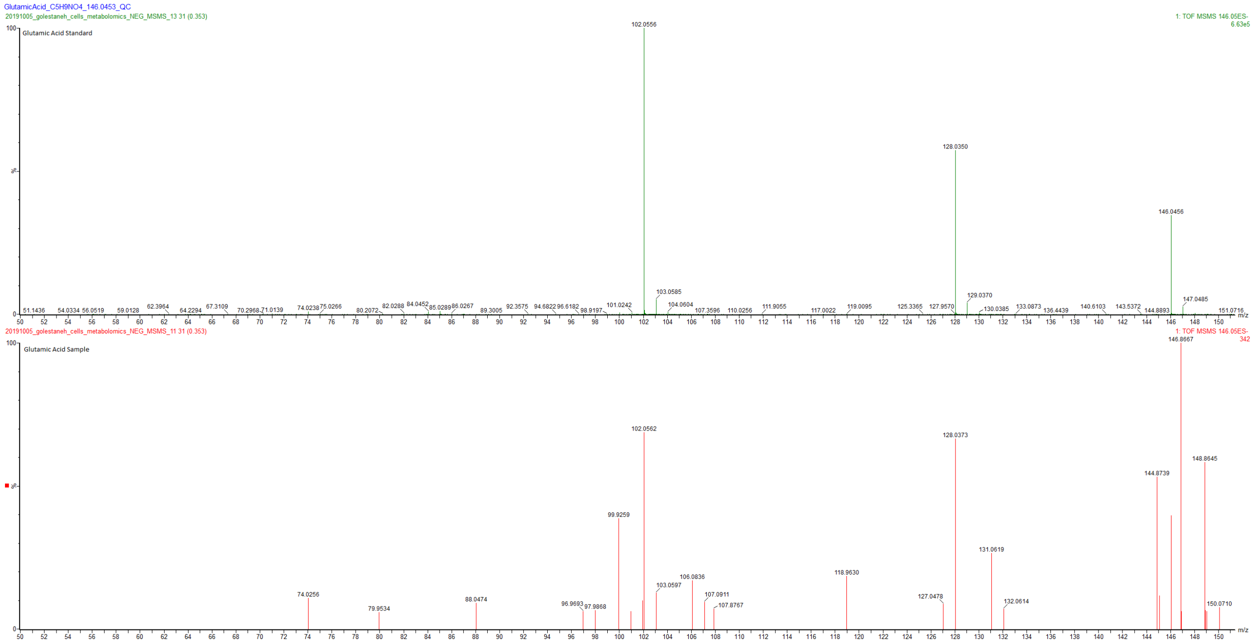

The figure shows the MS/MS fragmentation patterns of the m/z 146.0453 in the Glutamic Acid standard (top panel) and in the pooled QC sample (bottom panel).

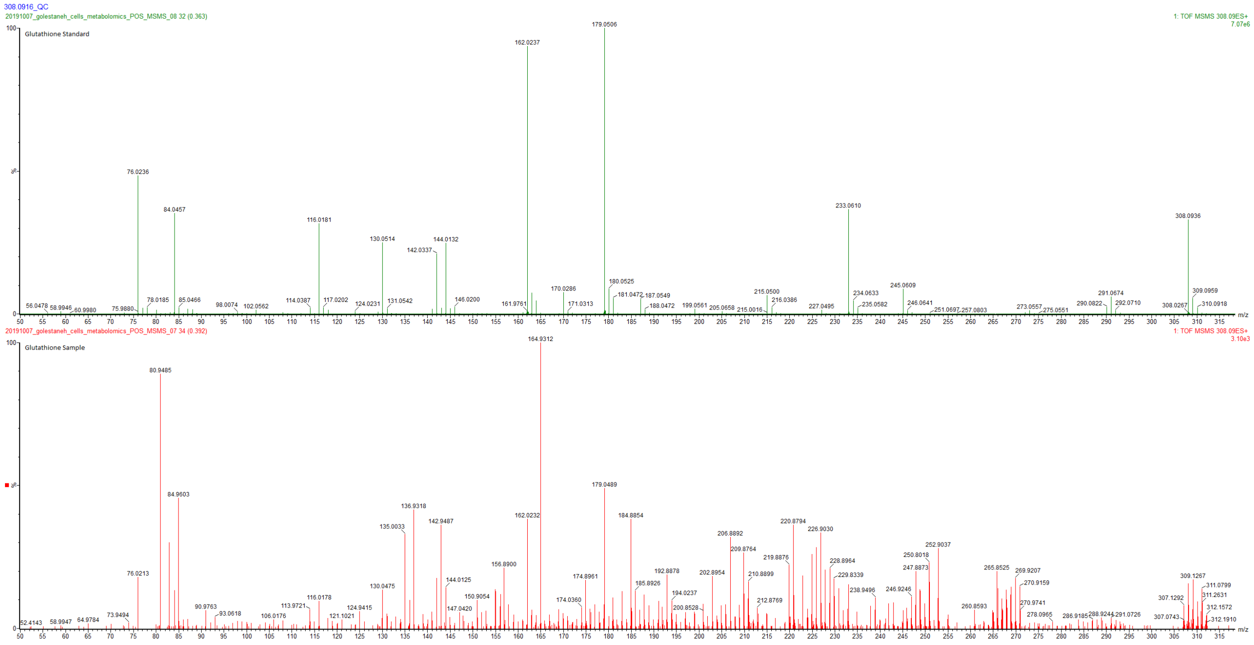

The figure shows the MS/MS fragmentation patterns of the m/z 308.0916 in the Glutathione standard (top panel) and in the pooled QC sample (bottom panel).The identifications for the compounds of interest were validated by UPLC-QTOF-MS/MS using a ramping collision energy. The compounds were validated by running MS/MS on each m/z a alongside standard.
